# 25(OH)D-but not 1,25(OH)_2_D–Is an independent risk factor predicting graft loss in stable kidney transplant recipients

**DOI:** 10.3389/fmed.2023.1141646

**Published:** 2023-04-20

**Authors:** Shufei Zeng, Yide Yang, Shuping Li, Carl-Friedrich Hocher, Chang Chu, Ziqiang Wang, Zhihua Zheng, Bernhard K. Krämer, Berthold Hocher

**Affiliations:** ^1^Division of Nephrology, Nanfang Hospital, Southern Medical University, Guangzhou, China; ^2^Key Laboratory of Molecular Epidemiology of Hunan Province, Changsha, China; ^3^Department of Nephrology, Center of Kidney and Urology, The Seventh Affiliated Hospital, Sun Yat-sen University, Shenzhen, China; ^4^Fifth Department of Medicine (Nephrology/Endocrinology/Rheumatology/Pneumonology), University Medical Centre Mannheim, University of Heidelberg, Heidelberg, Germany; ^5^Klinik für Innere Medizin, Bundeswehrkrankenhaus Berlin, Berlin, Germany; ^6^Department of Nephrology, The First Affiliated Hospital of Hainan Medical University, Haikou, China; ^7^European Center for Angioscience (ECAS), Medical Faculty Mannheim of the University of Heidelberg, Mannheim, Germany; ^8^Reproductive and Genetic Hospital of CITIC-Xiangya, Changsha, China; ^9^Key Laboratory of Study and Discovery of Small Targeted Molecules of Hunan Province, School of Medicine, Hunan Normal University, Changsha, China; ^10^Institute of Medical Diagnostics, IMD, Berlin, Germany

**Keywords:** kidney transplantation, all-cause mortality, graft loss, 25(OH)D, 1, 25(OH)_2_D

## Abstract

**Background:**

Vitamin D deficiency (VDD) or vitamin D insufficiency is common in kidney transplant recipients (KTRs). The impact of VDD on clinical outcomes in KTRs remain poorly defined and the most suitable marker for assessing vitamin D nutritional status in KTRs is unknown so far.

**Methods:**

We conducted a prospective study including 600 stable KTRs (367 men, 233 women) and a meta-analysis to pool existing evidence to determine whether 25(OH)D or 1,25(OH)_2_D predicted graft failure and all-cause mortality in stable KTRs.

**Results:**

Compared with a higher 25(OH)D concentration, a low concentration of 25(OH)D was a risk factor for graft failure (HR 0.946, 95% CI 0.912−0.981, *p* = 0.003), whereas 1,25 (OH)_2_D was not associated with the study end-point graft loss (HR 0.993, 95% CI 0.977−1.009, *p* = 0.402). No association was found between either 25(OH)D or 1,25 (OH)_2_D and all-cause mortality. We furthermore conducted a meta-analysis including 8 studies regarding the association between 25(OH)D or 1,25(OH)_2_D and graft failure or mortality, including our study. The meta-analysis results were consistent with our study in finding that lower 25(OH)D levels were significantly associated with the risk of graft failure (OR = 1.04, 95% CI: 1.01−1.07), but not associated with mortality (OR = 1.00, 95% CI: 0.98−1.03). Lower 1,25(OH)_2_D levels were not associated with the risk of graft failure (OR = 1.01, 95% CI: 0.99−1.02) and mortality (OR = 1.01, 95% CI: 0.99−1.02).

**Conclusion:**

Baseline 25(OH)D concentrations but not 1,25(OH)_2_D concentrations were independently and inversely associated with graft loss in adult KTRs.

## Introduction

Graft and patient survival rates after kidney transplantation (KT) have improved over the past decade. The death-censored graft survival rate has increased steadily in adults and pediatric recipients (1). Although sequential improvements in short-term graft patency were achieved, the advances were not accompanied by similar progress in long-term graft survival (2). Thus, there is an urgent yet unmet medical need to implement comprehensive strategies to improve long-term outcomes.

Vitamin D deficiency (VDD) or vitamin D insufficiency is common and conflicting results concerning VDD-associated graft failure and mortality were described in kidney transplant recipients (KTRs). Many studies showed that VDD, measured as 25-hydroxyvitamin D [25(OH)D] or 1,25-dihydroxyvitamin D [1,25(OH)_2_D], relates to kidney dysfunction in renal disease and graft loss in KTRs. An animal study demonstrated that VDD reduces renal function, worsens renovascular morphological features, and aggravates moderate chronic kidney disease (CKD) (3). Epidemiologic research observed that VDD is associated with an increased risk of CKD progression (4–7), while vitamin D analogs in two small clinical trials provided some indication of renoprotective effects by reducing proteinuria (8–10).

Moreover, other clinical studies revealed that when comparing KTR patients with deficient and insufficient vitamin D levels to KTR patients with sufficient vitamin D levels, the latter had better graft survival and overall survival outcomes (11–13).

However, some clinical studies reported conflicting results that VDD, whether measured as 25(OH)D or 1,25(OH)_2_D, is not associated with patient survival and graft loss in KTRs (14–17). Also, a recent meta-analysis (18) showed that early vitamin D deficiency was associated with a higher mortality rate after KT; graft loss was unaffected. However, the vitamin D status of the studies included was assessed by 25(OH)D only, focused on vitamin D status right after transplantation, and these studies were not corrected for the varying methods of vitamin D measurement.

Given the increasing knowledge of widespread deficiency of vitamin D with 10% of cases in North America and>80% in part of Asia (18) and the lack of clarity as to whether VDD is associated with clinical outcomes in KTRs and the most suitable marker for assessing vitamin D nutritional status, we conducted a prospective study and a meta-analysis to pool existing evidence and the current observational study to determine whether 25(OH)D or 1,25(OH)_2_D predicted graft failure and all-cause mortality in stable KTRs.

## Materials and methods

### Study population and design

This prospective cohort study comprises 600 KTRs who received a deceased kidney donation, which received a kidney transplant before October 15th, 2012, at the transplant clinic Charité-Mitte, Berlin, Germany. The clinical and research activities being reported are consistent with the Principles of the Declaration of Istanbul as outlined in the “Declaration of Istanbul on Organ Trafficking and Transplant Tourism.” Exclusion criteria: patients with an acute infection, malignancy, acute rejection, acute myocardial infarction, pulmonary edema, or heart failure at blood sampling. Patients were followed up for graft loss and all-cause mortality for 3 years. Loss of graft function was defined as the need for renal replacement therapy based on the judgment of the treating physicians. The protocol was approved by the ethics committee of Charité University Hospital (approval number 2012−327) under the Declaration of Helsinki. After the patients’ consent, clinical and laboratory data were collected. Patients’ blood was collected at the beginning of the study.

### Data sources and assays

Demographic data for recipients and donors (cold ischemia time, HLA mismatches, donor’s age, panel reactive antibodies, recipient’s age, sex, transplant survival, underlying kidney disease) were extracted from hospital records and the Euro-transplant records of the patients. Blood samples were collected from 600 KTRs during September and October 2012. EDTA was added to blood samples followed by centrifugation (4,500 rpm) for 20 min at 4°C, then plasma was collected and stored at −20°C until analysis. Laboratory parameters (including plasma 25(OH)D, 1,25(OH)_2_D, calcium, phosphorus, albumin, cholesterol, and creatinine) were measured by standardized laboratory techniques in the central clinical laboratory of the Charité Universitaetsmedizin Berlin, Germany.

### Statistical analysis

Data are presented as median (interquartile range) and number (percentage) for normally distributed and nominal data. A *p*-value of less than 0.05 (two-tailed) was considered statistically significant. Statistical analyses were performed using SPSS 20.0 for Windows (SPSS Inc.) and STATA 14 (StataCorp, College Station, TX, USA).

**FIGURE 1:**
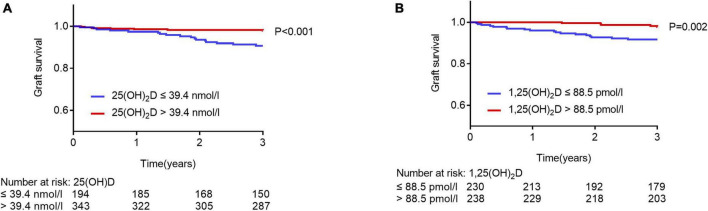
Graft survival rates in patients with higher and lower levels of plasma 25(OH)D or 1,25(OH)_2_D. Kaplan-Meier curves, according to plasma 25(OH)D and 1,25 (OH)_2_D for graft failure. Survival rates were compared with the log-rank test. **(A)** Blue lines, patients with plasma 25(OH)D levels of ≤ 39.4 nmol/l; red lines, patients with plasma 25(OH)D levels of > 39.4 nmol/l. **(B)** Blue lines, patients with plasma 1,25(OH)_2_D levels of ≤ 88.5 pmol/l; red lines, patients with plasma 1,25(OH)_2_D levels of > 88.5 pmol/l.

**FIGURE 2:**
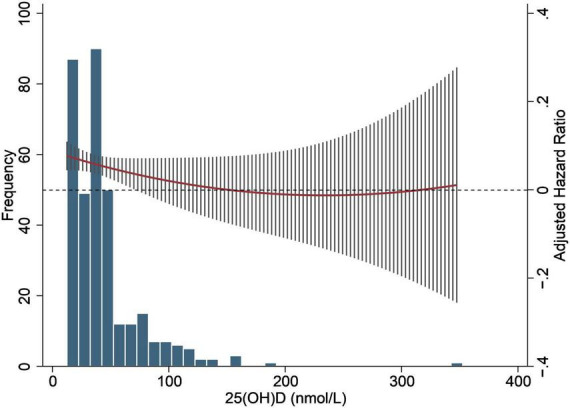
Estimated spline for the hazard ratio of graft failure with 25(OH)D in renal transplant recipients. Y axis represents spline smooth functions. Tick marks in X axis indicate distribution of observations. Dashed lines designate 95% confidence interval for smoothing functions.

The blood levels of 25(OH)D and 1,25(OH)_2_D were firstly analyzed using the area under the receiver operating characteristic (ROC) curve to obtain the optimal cut-off values, respectively (Supplementary Figure 1). Since the histograms of all parameters do not suggest multimodal distribution and the normality tests indicate normal distributions, plasma levels of all parameters in patients with 25(OH)D above or below the cutoff-value were compared using the independent *T*-test in Table 1. Furthermore, using these cut-off values, time-to-event analyses were performed with the log-rank test using the Kaplan-Meier method (Figure 1). In addition, multivariable-adjusted graft failure analyses were performed using Cox proportional hazards regression models (Tables 2, 3). Based on the following confounding factors: patient age, sex, donor age, cold ischemia time, urine protein, time on dialysis, eGFR, plasma calcium, phosphate, iPTH, hemoglobin, immunosuppressants and HLA mismatches, several models were built by adding 25(OH)D, 1,25(OH)_2_D and then both, respectively, to analyze which is the independent risk factor for graft loss in KTRs. Pearson’s correlation analysis was conducted to illustrate the correlation between the two forms of vitamin D and eGFR (Supplementary Figure 2). Furthermore, the 25(OH)D curves with hazard ratio were fitted for graft loss in the generalized additive model (GAM) (Figure 2).

**TABLE 1:** Patient characteristics of adult kidney transplant recipients (*n* = 600).

	All (*n* = 600)	25(OH)D ≤ 39.4 nmol/l	25(OH)D > 39.4 nmol/l	*P*
*N*	600	202	347	
Age at study **e**ntry (years)	55.0 (22.0)	56.0 (26.0)	54.0 (22.0)	0.742
Sex (female/male)	233 f/367 m	78 f/124 m	133 f/214 m	
Donor age (years)	52.0 (23.0)	50.0 (25.8)	53.5 (20.0)	0.231
Time on dialysis (months)	47.5 (59.0)	40.0 (54.0)	50.0 (56.0)	0.038
Time post-transplantation (months)	60.0 (77.0)	69.5 (86.0)	58.0 (70.0)	0.399
Cold ischemia time (hours)	9.8 (8.9)	9.8 (11.2)	10.4 (7.9)	0.420
eGFR (mL/min/1.73 m^2^)	43.0 (26.0)	45.0 (24.0)	41.0 (26.8)	0.424
Hemoglobin (g/dl)	12.7 (2.5)	13.1 (2.8)	12.8 (2.6)	0.453
Plasma albumin (g/dl)	4.5 (0.5)	4.5 (0.6)	4.5 (0.4)	0.225
Plasma creatinine (mg/dl)	1.57 (0.74)	1.5 (0.7)	1.6 (0.8)	0.879
Total cholesterol (mg/dl)	219.0 (77.5)	217.0 (85.0)	220.0 (70.3)	0.520
HbA1c (%)	5.8 (0.8)	5.8 (0.8)	5.8 (0.8)	0.447
Plasma calcium (mmol/L)	2.5 (0.2)	2.5 (0.2)	2.4 (0.2)	0.007
Plasma phosphate (mmol/L)	0.8 (0.3)	0.9 (0.3)	0.8 (0.3)	0.804
Fasting blood glucose (mg/dl)	88.0 (32.3)	87.0 (29.8)	89.0 (34.5)	0.518
Urinary protein (mg/24 h)	166.5 (209.0)	163.0 (298.0)	169.0 (185.0)	0.173
Plasma iPTH (pg/ml)	85.8 (91.9)	90.2 (129.9)	81.0 (89.1)	0.001
Plasma 25(OH)D (nmol/L)	52.1 (43.6)	12.5 (20.0)	64.3 (31.1)	<0.001
Plasma 1,25(OH)_2_D (pmol/L)	90.5 (72.8)	82.5 (75.3)	92.0 (71.5)	0.014
1,25(OH)_2_D/25(OH)D (*10^–3^)	1.9 (2.3)	4.4 (3.8)	1.4 (1.2)	<0.001
**Immunosuppressant**				
Cyclosporin A	193 (36.4%)	74 (36.6%)	116 (33.4%)	
Tacrolimus	238 (44.9%)	81 (40.1%)	139 (40.1%)	
Everolimus	82 (15.5%)	31 (15.3%)	62 (17.9%)	
Combined medication	17 (3.2%)	6 (3.0%)	11 (3.2%)	
HLA mismatches				
**HLA-A**				
0 mismatch	233 (38.8%)	77 (38.1%)	137 (39.5%)	
1 mismatch	272 (45.3%)	90 (44.6%)	158 (45.5%)	
2 mismatches	95 (15.8%)	35 (17.3%)	52 (15.0%)	
**HLA-B**				
0 mismatch	174 (29%)	55 (27.2%)	101 (29.1%)	
1 mismatch	270 (45%)	89 (44.1%)	160 (46.1%)	
2 mismatches	156 (26%)	58 (28.7%)	86 (24.8%)	
**HLA-DR**				
0 mismatch	210 (35%)	70 (34.7%)	121 (34.9%)	
1 mismatch	289 (48.2%)	101 (50.0%)	167 (48.1%)	
2 mismatches	101 (16.8%)	31 (15.3%)	59 (17.0%)	

Values are presented as median (interquartile range) or n (%). eGFR, estimated glomerular filtration rate; HbA1c, hemoglobin A1c; iPTH, intact parathyroid hormone; 25(OH)D, 25-hydroxyvitamin D; 1,25(OH)_2_D, 1,25 dihydroxy vitamin D; HLA, human leukocyte antigens.

**TABLE 2:** Cox proportional hazards analysis of 25(OH)D or 1,25(OH)_2_D and the relevant factors for graft loss in renal transplant recipients.

		25(OH)D			1,25(OH)_2_D	
	**HR**	**95% CI**	***P***	**HR**	**95% CI**	***P***
Model A	0.968	0.951−0.985	<0.001	0.987	0.976−0.997	0.011
Model B	0.962	0.943−0.981	<0.001	0.985	0.974−0.996	0.007
Model C	0.959	0.933−0.985	0.002	0.983	0.969−0.998	0.024
Model D	0.946	0.912−0.981	0.003	0.993	0.977−1.009	0.402
Model E	0.947	0.911−0.985	0.006	0.989	0.969−1.009	0.274

Multiple proportional hazards regression analyses (Cox regression; enter). Patients were followed for graft loss for 3 years. Model A: crude. Model B: adjusted for patient age, sex, and donor age. Model C: as model B and additionally adjusted for cold ischemia time, urine protein, time on dialysis, plasma calcium, iPTH, hemoglobin. Model D: as model C and additionally adjusted for eGFR and plasma phosphate. Model E: as model D and additionally adjusted for cyclosporin A, tacrolimus, everolimus and mismatches of HLA-A, HLA-B, HLA-DR.

**TABLE 3:** Cox proportional hazards analysis of potential bio-marker predicted graft failure of renal transplant patients forward likelihood ratio model (*n* = 600).

	HR	95% CI	*P*
Sex	0.151	0.037−0.620	0.009
Urinary protein	1.001	1.000−1.001	<0.001
Plasma phosphate	58.424	10.572−322.880	<0.001
Plasma hemoglobin	0.598	0.424−0.842	0.003
Plasma 25(OH)D	0.943	0.912−0.975	<0.001

25(OH)D, 1,25(OH)_2_D + patient age, sex, donor age, cold ischemia time, urine protein, time on dialysis, eGFR, Ca, PO4, PTH, and Hb being the independent variable.

For the association between 25(OH)D, or 1,25(OH)_2_D and the risk of graft failure or mortality, we further conducted a meta-analysis to estimate the pooled estimates (HR). First, we searched PubMed electronically, using terms relating to vitamin D [e.g., vitamin D or 25-hydroxyvitamin D or 1,25-dihydroxyvitamin D or 1,25(OH)_2_D or 25(OH)D], kidney transplantation (e.g., Kidney transplantation or renal transplantation or kidney transplant or renal transplant) and (graft failure or graft loss). The inclusion criteria of this meta-analysis were: original paper; follow-up studies with 1,25(OH)_2_D or 25(OH)D data available; outcome included graft loss or mortality; published before December 31st, 2021; the language of the study was English. Exclusion criteria were: animal study or review paper.

After screening the papers, we included seven studies that met the inclusion criteria for our meta-analysis to calculate the pooled HR of the association between 1,25(OH)_2_D or 25(OH)D and the risk of graft failure or mortality. Fixed effects models were used for studies without significant heterogeneity, while random-effects models were used for studies with significant heterogeneity. RevMan Manager version 5.4 software^1^ was used for the present meta-analysis.

## Results

### Study population

The baseline characteristics for 600 KTRs consisting of 233 men and 367 women aged 20–87 years are shown in Table 1, which contains both clinical and laboratory parameters. After 3 years of follow-up, 38 patients showed graft failure, and 65 died in this observation period.

### Association of vitamin D status and all-cause mortality

In order to separate the study population, 1,25(OH)_2_D and 25(OH)D were firstly analyzed using ROC analysis for all-cause mortality to obtain the cut-off values [1,25(OH)_2_D 83.5 pmol/l; 25(OH)D 52.35 nmol/l]. No association was found between two forms of vitamin D and all-cause mortality using the Kaplan-Meier survival curve [1,25(OH)_2_D, *p* = 0.385; 25(OH)D, *p* = 0.616; data do not show]. Furthermore, univariable Cox regression analysis indicated that none of the two forms of vitamin D is associated with all-cause mortality [1,25(OH)_2_D, HR 1.001, 95% CI 0.996−1.006, *p* = 0.688; 25(OH)D, HR 1.004, 95% CI 0.998−1.011, *p* = 0.154; data do not show].

### Association of vitamin D status and graft loss, and eGFR

We separated the study population with optimal cut-off values of 1,25(OH)_2_D and 25(OH)D, respectively, using a ROC analysis for graft loss (Supplementary Figure 1). In a Kaplan-Meier survival curve in patients after kidney transplantation, 25(OH)D concentrations above 39.4 nmol/l were significantly associated with graft loss (Figure 1, *p* < 0.001, log-rank test), as well as 1,25(OH)_2_D concentrations above 88.5 pmol/l were associated with graft loss (*p* = 0.002, log-rank test). On the contrary, the ratio of 1,25 (OH)_2_D and 25(OH)D was not associated with graft loss (*p* = 0.531, log-rank test, data do not show).

We analyzed the impact of 1,25(OH)_2_D, as well as 25(OH)D in KTRs using Cox regression models by gradually adding confounding factors (Table 2). In univariable Cox analyses, both 1,25(OH)_2_D and 25(OH)D were significantly associated with graft loss (Model A, HR 0.987, 95% CI 0.976−0.997, *p* = 0.011; HR 0.968, 95% CI 0.951−0.985, *p* < 0.001). The association between 25(OH)D and graft loss remained significant after adjustment for known confounders factors (Model D, HR 0.946, 95% CI 0.912−0.981, *p* = 0.003). However, 1,25 (OH)2D was no longer significantly correlated to graft loss after adding an adjustment for eGFR and plasma phosphate (Model D, HR 0.993, 95% CI 0.977−1.009, *p* = 0.402). In addition, we build model E based on model D and additionally adjusted for cyclosporin A, tacrolimus, everolimus and mismatches of HLA-A, HLA-B, HLA-DR, and the association between 25(OH)D and graft loss remained significant (model E, HR 0.947, 95% CI 0.911−0.985, *p* = 0.006). Moreover, we also performed Cox regression models using the forward likelihood ratio method. This approach likewise showed that a low concentration of plasma 25(OH)D (Table 3, HR 0.943, 95% CI 0.912−0.975, *p* < 0.001) was a risk factor for graft failure, whereas again, 1,25 (OH)2D concentrations were not associated with the study end-point graft loss.

Supplementary Figure 2 showed that eGFR was positively correlated with 1,25(OH)_2_D plasma concentration but not with 25(OH)D plasma concentration in KTRs. Furthermore, Figure 2 reveals that graft loss hazard ratios (HRs) were inversely associated with 25(OH)D in a non-linear model.

### Meta-analysis

**FIGURE 3:**
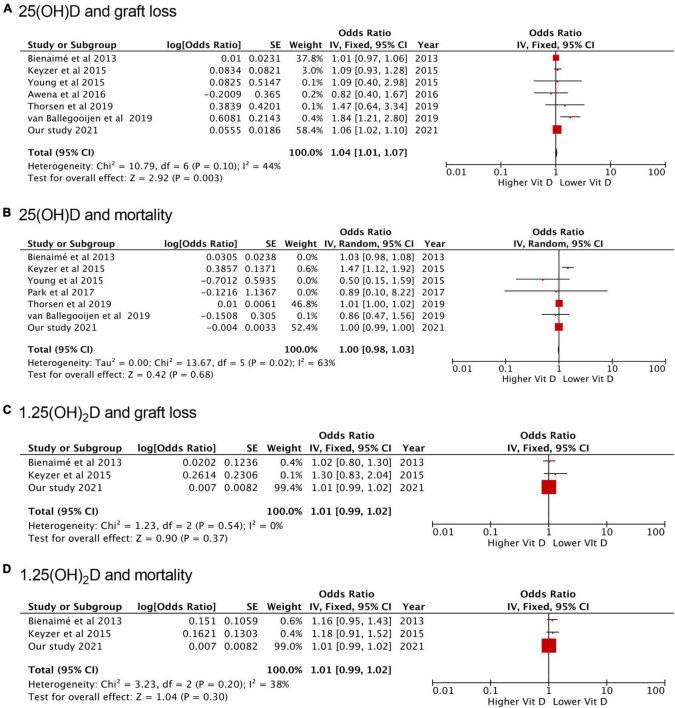
Forest plots of the included studies evaluating the association between vitamin D and death and graft loss. **(A)** 25(OH)D and graft loss; **(B)** 25(OH)D and mortality; **(C)** 1,25(OH)_2_D and graft loss; **(D)** 1,25(OH)_2_D and mortality. SE, standard error; IV, inverse-variance; CI, confidence interval.

We performed a meta-analysis to assess the association between 1,25(OH)_2_D or 25(OH)D and graft failure or mortality. Lower 25(OH)D levels were significantly associated with the risk of graft failure (OR = 1.04, 95% CI: 1.01−1.07) but not associated with mortality (OR = 1.00, 95% CI: 0.98−1.03) (Figure 3). Lower 1,25(OH)_2_D levels were not associated with the risk of graft failure (OR = 1.01, 95% CI: 0.99−1.02) and mortality (OR = 1.01, 95% CI: 0.99−1.02) (Figure 3).

## Discussion

We performed an observational cohort study and meta-analysis to investigate the effect of low 25(OH)D and 1,25(OH)_2_D levels measured in stable KTRs on graft failure and all-cause mortality. This meta-analysis is the first to summarize the available evidence on the long-term outcomes of 25(OH)D and 1,25(OH)_2_D in KTRs head-to-head in one study. The cohort study and meta-analysis results were consistent in finding that baseline 25(OH)D concentrations were independently and inversely associated with graft loss regardless of known confounding factors, whereas 25(OH)D concentrations were not associated with mortality in adult KTRs. In addition, no association of 1,25(OH)_2_D with mortality and graft failure was found in adult KTRs.

The patient characteristics indicated that the cohort study population was representative of a typical European post-transplant cohort (19). We observed that the incidence rate of renal graft loss was 6.33% (38/600) after a follow-up of 3 years, in line with the average in other centers (2). Therefore, our results are generally applicable. In addition to our cohort study, the meta-analysis included seven clinical studies published on the prognostic impact of vitamin D levels in KTRs with comparable patient characteristics to the study population in our study (Table 4) (11–17).

**TABLE 4:** Information clinical studies analyzing the correlation between vitamin D levels and survival in renal transplant recipients.

	Region	Time and journal	*N*	Sex (M/F %)	Indicator	Cutoff	Blood collection time	Follow	Results
Bienaime et al. (14)	France	J Am Soc Nephrol. 2013	634	58.7/41.3	25(OH)D; 1,25(OH)_2_D	No	3 months	48.6 months (median)	Low 25(OH)D or 1,25(OH)D concentrations 3 months after transplantation did not predict early death or graft loss.
Thorsen et al. (11)	Norway	Clinical transplantation, 2019	762	67.6/32.4	25(OH)D	30 nmol/L and 50 nmol/L	10 week after transplantation	82 months (median)	Long-term graft and patient survival were better in recipients with vitamin D sufficiency 10 weeks post-transplant compared with those with vitamin D deficiency and insufficiency.
Keyzer et al. (12)	Netherlands	The Journal of Clinical Endocrinology and Metabolism, 2015	435	51/49	25(OH)D; 1,25(OH)_2_D	No	6 years (median) after transplantation	7 years (median)	Low 25(OH)D is independently associated with an increased risk of all-cause mortality and 25(OH)D<12 ng/ml with a rapid eGFR decline in stable KTR. The association of low 1,25(OH)_2_D with mortality or graft failure depends on renal function.
Kwon et al. (15)	Korea	Medicine (Baltimore). 2015	410	63.9/36.1	25(OH)D	10 ng/mL	Within 2 weeks before kidney transplantation	7.3 years (median)	25(OH)D deficiency was not significantly associated with patient mortality and graft failure.
van Ballegooijen et al. (13)	Netherlands	Nephrol Dial Transplant. 2020	461	53.1/46.9	25(OH)D	50 nmol/L	At transplantation	6.1 years (median)	Combined vitamins D and K deficiency are highly prevalent and are associated with increased mortality and graft failure risk compared with high vitamins D and K status.
Le Fur et al. (16)	France	Transpl Int. 2016	444	60.6/39.4	25(OH)D	10 and 30 ng/mL	At transplantation	12 months	25(OH)D deficiency was not significantly associated with patient–graft survival.
Park et al. (17)	Korea	Korean J Intern Med. 2017	164	70.70/29.3	25(OH)D	20 ng/mL	Within 2 week before kidney transplant	24.8 months	The frequencies of allograft failure and patient death did not significantly differ between patients with the low and high vitamin D.
Our study	Germany		600	61.2/38.8	25(OH)D; 1,25(OH)_2_D	No	average 7 years after transplantation	3 years	25(OH)D- but not 1,25(OH)_2_D- is an independent risk factor predicting graft loss in stable renal transplant recipients

Our cohort study and meta-analysis consistently showed that 25(OH)D concentrations were associated with increased graft loss rates but not mortality. Although some of the previous clinical studies (11–13) suggested a relationship between vitamin D levels and all-cause mortality, we did not find any benefit in patient survival from higher concentrations of 25(OH). This discrepancy may be due to the small sample size of previous studies. They were most likely underpowered for this end-point. Hence, our metanalysis is helpful by combining the evidence from all published studies, including our data, and indicates no mortality effect of either 25(OH)D or 1,25(OH)_2_D.

Our finding that 25(OH)D is associated with graft loss fits very well with published preclinical studies in the field. VDD deficiency was linked to an impaired kidney function in a CKD animal model (3) and associated with increased graft failure risk in experimental KTRs (11–13). It has been confirmed that vitamin D analogs inhibit kidney fibrosis with potential renoprotective activity in a cyclosporine-induced rat model of CKD (20). Renal vitamin D receptor binding to nuclear response elements is reduced in rats with incipient renal failure (21).

Moreover, the administration of vitamin D is beneficial in experimental transplantation models. 1,25(OH)_2_D administration prevented acute rejection and prolonged survival in the ACI to Lewis rat renal transplant model (22) and also prevented chronic allograft nephropathy in the Fisher 344 to Lewis rat renal transplantation model (23). A close association between VDD and graft loss was also reported in liver transplant recipients. Martucci et al. (24) showed that after orthotopic liver transplantation, incomplete graft recovery was associated with lower vitamin D on postoperative day (POD) 28 (OR: 0.84; CI 95%: 0.73−0.97; *P* = 0.014), indicating that the value of vitamin D on POD28 had a strong association with graft function.

The deficiency of 25(OH)D, but not 1,25(OH)_2_D, in blood circulation is an independent risk factor for diminished allograft survival in our cohort study and meta-analysis. It may be that the stability of 25(OH)D in the circulation makes it more representative of the patient’s vitamin D status. It is known that 25(OH)D has a higher affinity for vitamin D’s transport protein than 1,25(OH)_2_D but a lower affinity for specific VDR (25). Because of these features, 25(OH)D is considered a transfer form rather than a biological effector *in vivo*. Compared to 1,25(OH)_2_D, 25(OH)D in the peripheral circulation usually has a higher concentration, lower affinity, and a longer half-life (26) describing better the average vitamin status and or vitamin D substitution than 1,25(OH)_2_D and may thus be better linked to outcomes such as graft loss.

The association between low 1,25(OH)_2_D and graft failure was seen in our cohort and was dependent on renal function. This association is not unexpected since the 1α-hydroxylase of the kidney, i.e., the enzyme that converts 25(OH)D into 1,25(OH)_2_D, is damaged simultaneously with the deterioration of renal function.

1,25(OH)_2_D is identified as a VD form with relatively short half-life that is also influenced by adrenocorticotropic hormone (27) and sex hormone (28). Furthermore, 1,25(OH)_2_D has been shown to reduce glomerulosclerosis and urinary albumin excretion in progressive glomerular damage (25). Furthermore, it has been demonstrated that 1,25(OH)_2_D has anti-proliferative properties for glomeruli (29) and has a renal protective effect by targeting podocytes (30, 31). Therefore, in the case of chronic kidney damage, it makes little sense to correlate graft loss with 1,25(OH)_2_D concentrations alone; instead, it is more reasonable to adjust the association of low 1,25(OH)_2_D concentrations with graft failure and mortality for impaired renal function (12). This could be why 25(OH)D, rather than 1,25(OH)_2_D, is a better marker of VD status in this patients cohort and can independently predict graft loss.

Given the high prevalence of vitamin D deficiency (VDD) in renal transplant patients and that VDD is treatable, a target concentration of 25(OH)D needs to be determined. The generalized additive model (GAM) (see Figure 2) shows that in the patients with 25(OH)D levels below 150 nmol/L, the hazard ratio decreases with an increase of 25 (OH)D levels in stable KTRs. A recent guideline suggested that vitamin D exceeding 250 nmol/L is deemed a “risk” of vitamin D toxicity (32). In Figure 2, the curve of 25(OH)D with hazard ratio in generalized additive model (GAM), the levels of 310 nmol/L is above zero, and the curve’s trend increases with a numerical increase of 25(OH)D. Since only a very small number of patients had levels of 25(OH)D that exceed the level of possible adverse effects, we cannot make any firm conclusions if an unlimited increase in 25(OH)D may increase the risk ratio of graft failure and at what precise concentration of 25(OH)D this may occur. Nowadays, no clear criteria have been applied to define the deficiency/insufficiency status (33). The bone centered guidelines recommend a target 25(OH)D concentration of at least 20 ng/mL (50 nmol/L), while the guidelines that focus on the pleiotropic effect of vitamin D recommend a higher threshold of 25(OH)D concentration of 30 ng/mL (75 nmol/L). Despite that, it seems evident that most KTRs have moderately or even severely decreased levels of native vitamin D (34, 35). Vitamin D deficiency is a widespread global health problem (36). Since 74% of KTR’s sample was below 30 ng/mL (75 nmol/L) in our cohort, 25(OH)D Vitamin D deficiency/insufficiency in KTRs is more frequent and severe than in the general population. Most KTRs require an additional replenishment of vitamin D. The optimal target interval of vitamin D levels in the healthy general population may not be appropriate for KTRs, and the 25 (OH)D level must be much higher than 50 nmol/mL effectively improve the clinical outcome of KTRs.

Several limitations of our cohort study warrant consideration:

1. Although we have eliminated several potential known confounding factors, we cannot exclude the possibility of remaining confounding, as our study had an observational character.

2. Since barely any patients with the levels of 25(OH)D exceed the level of possible adverse effects [i.e., 25(OH)D levels>250 nmol/L], we do not know what concentration of 25 (OH) D may have an adverse effect.

This meta-analysis has several strengths and limitations:

1. All included studies were observational studies, and 25% were retrospective.

2. The studies assessed 25(OH)D levels at different time points, from 2 weeks before transplant to 6 months after. In addition, the use of different cut-off values may increase heterogeneity among studies.

3. Although early VDD was confirmed as a risk factor for inferior outcomes, studies focusing on the effect of vitamin D supplements on transplant outcomes are lacking.

4. Finally, just total but not the bioactive free form of 25(OH)D was measured. This, however, might be especially important in patients with CKD (33, 37–40).

In conclusion, 25(OH)D was independently associated with graft loss in adult KTRs. The lower optimal range of 25(OH)D to prevent renal graft loss in KTRs seems to be 50 nmol/L. The association of low 1,25(OH)_2_D with graft failure depends on renal function. Vitamin D deficiency/insufficiency in KTRs is widespread and might be a preventable risk factor for graft loss.

## Data availability statement

The raw data supporting the conclusions of this article will be made available by the authors, without undue reservation.

## Ethics statement

The protocol was approved by the Ethics Committee of Charité University Hospital (approval number 2012−327) under the Declaration of Helsinki. The patients/participants provided their written informed consent to participate in this study.

## Author contributions

BH initiated this study, finalized and critically reviewed the manuscript, and approved the final version. SZ wrote the manuscript and conducted the literature search of the meta-analysis. SL drafted the Figure 2. YY drafted the Figure 3, performed the data extraction, quality assessment, and statistical analysis of the meta-analysis. SL, C-FH, CC, and ZW edited the manuscript and collected data. ZZ, BK, and BH reviewed the manuscript. All authors contributed to the article and approved the submitted version.

## Footnotes

1
http://www.cc-ims.net/revman
