# Supplementary material for: 25(OH)D-but not 1,25(OH)2D–Is an independent risk factor predicting graft loss in stable kidney transplant recipients
Source: Front Med (Lausanne). 2023 Apr 20;10:1141646. doi: 10.3389/fmed.2023.1141646 (PMC10156982; doi:10.3389/fmed.2023.1141646)
Supplement: Supplementary file 1 [file Image_1.pdf]

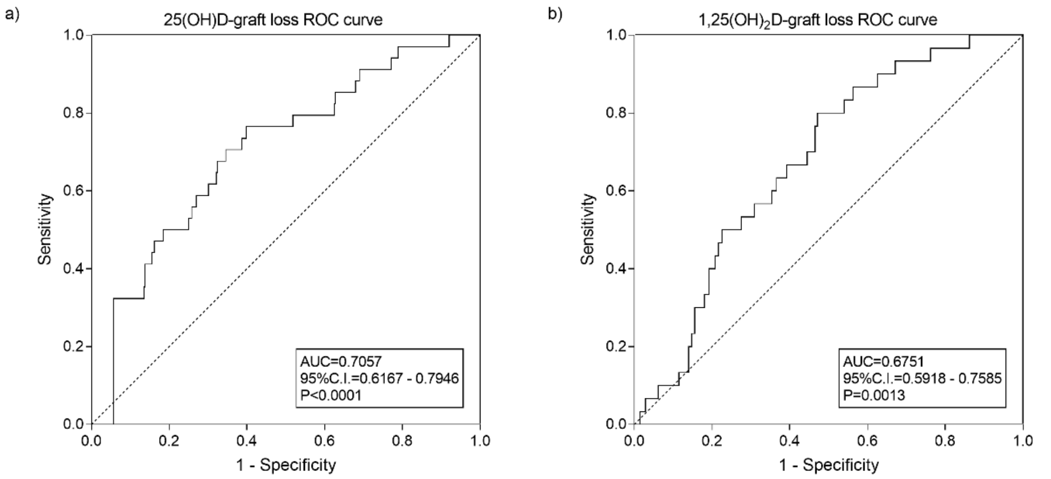

Supplementary Figure 1. ROC curve of plasma 25(OH)D and 1,25(OH)$_2$D as test variable and graft loss until follow up as state variable.
