# Supplementary material for: 25(OH)D-but not 1,25(OH)2D–Is an independent risk factor predicting graft loss in stable kidney transplant recipients
Source: Front Med (Lausanne). 2023 Apr 20;10:1141646. doi: 10.3389/fmed.2023.1141646 (PMC10156982; doi:10.3389/fmed.2023.1141646)
Supplement: Supplementary file 2 [file Image_2.pdf]

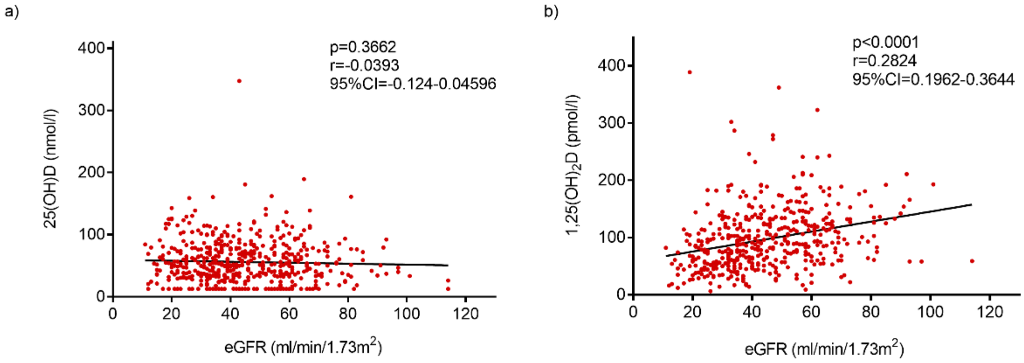

Supplementary Figure 2. Association of eGFR and 25(OH)D and 1,25(OH)$_2$D respectively.

eGFR was positively correlated with 1,25(OH)$_2$D plasma concentration, but not with 25(OH)D in renal transplant recipients.
